# Supplementary material for: High-throughput widefield fluorescence imaging of 3D samples using deep learning for 2D projection image restoration
Source: PLoS One. 2022 May 19;17(5):e0264241. doi: 10.1371/journal.pone.0264241 (PMC9119453; doi:10.1371/journal.pone.0264241)
Supplement: Attachment — Submitted filename: Reponse-to-reviewers.pdf [file pone.0264241.s001.pdf]

Detailed Response to Reviewers - TMI-2021-1480

April 27, 2022

Dear Sathishkumar V E

Thank you for giving us the opportunity to re-submit our manuscript "High-throughput wide-field fluorescence imaging of 3D samples using deep learning for 2D projection image restoration" for publication in *PLOS One*. We appreciate the time and effort that you and the reviewers have spent reading and providing valuable feedback on our manuscript. We are grateful for the insights, which made significant improvements to our paper. We have incorporated most of the suggestions made by the reviewers and they are highlighted within the manuscript. Please see point-by-point responses to yours and the reviewers' comments, highlighted in blue.

1 Point-by-point response to comments

1.1 Associate Editor's Comments

Associate Editor:

Comments to the Author:

When submitting your revision, we need you to address these additional requirements. 1. Please ensure that your manuscript meets PLOS ONE's style requirements, including those for file naming. The PLOS ONE style templates can be found at `https://journals.plos.org/plosone/s/file?id=wjVg/PLOSOne_formatting_sample_main_body.pdf` and `https://journals.plos.org/plosone/s/file?id=ba62/PLOSOne_formatting_sample_title_authors_affiliations.pdf`
Author response: We have updated the names of the figure files, formatting regarding authorship list, changed from Fig. to Fig in the manuscript, moved figures to appear after the paragraph they are first mentioned in.

2. In your Methods section, please include information on the source or supplier of the cell lines used.
Author response: Thank you for this suggestion. We have added the information in **bold** in the paragraph directly under the Experiments heading:
"To establish proof of concept across multiple biological protocols, we tested our proposed workflow on three common protocols for culturing live 3D tumor spheroids, each of which present different

imaging challenges. **Four common immortalized cancer cell lines were used across the experiments: A549 (lung cancer), MCF-7 (breast cancer), MDA-MB-231 (breast cancer) and SK-OV-3 (ovarian cancer). Commercially available cell lines stably expressing a nuclear-restricted mKate2 FP were used for A549 and MCF-7, while standard SK-OV-3 (EACC) and MDA-MB-231 (ATCC) cell lines were purchased and stably transfected with the Incucyte® NucLight Red lentivirus reagent (EF1 Alpha Promoter; Sartorius) per manufacturer's instructions.** Because the FP is nuclear restricted, it can be used to measure cell viability, i.e. a loss of viability results in a compromised nuclear membrane to cause a loss of fluorescence. The sections below describe these three protocols."

1.2 General questions

1. Is the manuscript technically sound, and do the data support the conclusions?

The manuscript must describe a technically sound piece of scientific research with data that supports the conclusions. Experiments must have been conducted rigorously, with appropriate controls, replication, and sample sizes. The conclusions must be drawn appropriately based on the data presented.

Reviewer 1: Yes
Reviewer 2: Partly

2. Has the statistical analysis been performed appropriately and rigorously?

Reviewer 1: Yes
Reviewer 2: No
Author response: In response to these questions 1 and 2, we have added two additional metrics (MS-SSIM and MSE) to the results to further support our conclusions. During the process of calculating these new metrics, we also found a bug in our metric calculation script for the single spheroid data and have updated the metrics accordingly.

3. Have the authors made all data underlying the findings in their manuscript fully available?

The PLOS Data policy requires authors to make all data underlying the findings described in their manuscript fully available without restriction, with rare exception (please refer to the Data Availability Statement in the manuscript PDF file). The data should be provided as part of the manuscript or its supporting information, or deposited to a public repository. For example, in addition to summary statistics, the data points behind means, medians and variance measures should be available. If there are restrictions on publicly sharing data—e.g. participant privacy or use of data from a third party—those must be specified.

Reviewer 1: No
Reviewer 2: No
Author response: Thank you for noticing this. While we previously only included the repository information in our submission letter, we have now added a sentence to the Experiment section where we point the reader to a link on figshare where the data is available (`https://figshare.com/projects/Dataset_of_fluorecent_3D-samples_projected_to_2D/126629`).

4. Is the manuscript presented in an intelligible fashion and written in standard English?

PLOS ONE does not copyedit accepted manuscripts, so the language in submitted articles must be clear, correct, and unambiguous. Any typographical or grammatical errors should be corrected at revision, so please note any specific errors here.

Reviewer 1: Yes
Reviewer 2: No
Author response: We noted a number of grammatical mistakes and typos in our previous submission, and have gone through and corrected them to the best of our ability. We hope that our manuscript will now fulfill the language requirements for the journal.

1.3 Reviewer 1

1. Comparison study is not provided.
Author response: We have added a number of additional references in the Introduction (see response to comment 1 from Reviewer 2) to studies that are also combining deep learning methods with fluorescent imaging. A direct comparison study is hard to come by since our acquiring method of z-sweep without hardware modifications or several z-slices has not been done before. We have made this clearer by stating that this gap exists in the field of fluorescent imaging of complex 3D samples.

2. As you mentioned that 96 images were used for research. It is not available in public.
Author response: Thank you for acknowledging this. We have added a sentence in the Experiment section where we point the reader figshare `https://figshare.com/projects/Dataset_of_fluorecent_3D-samples_projected_to_2D/126629` where the data is available.

3. The deep learning model techniques, results need to compare.
Author response: We have expanded on the metrics in Table 1 to further compare the deep learning models results. Also, as mentioned in comment 1 above, we have added references containing similar works. Although no direct comparison study could be found.

4. Provide in detail about OSA-CGAN.
Author response: We thank you for this comment, as it made us realized that we did properly define the OSA-CGAN architecture. This has been remedied and is now addressed in the whole last paragraph in the **Conditional Generative Averserial Network** section. We hope this addition more clearly and explicitly defines both the OSA-CGAN and U-Net models:
"We investigated the use of two different generators for our CGAN architectures, an U-Net generator and an OSA-U-Net variant, both described in section Image Generator. The corresponding architectures are refereed to as CGAN (U-Net generator) and OSA-CGAN (OSA-U-Net generator) respectively and both versions uses the PatchGAN discriminator described in above section."

5. Overall the idea was good.
Author response: Thank you!

1.4 Reviewer 2

Comments to the Author

Widefield fluorescence microscopy is commonly used to study biological phenomena. However, fluorescent microscopic imaging of complex 3D samples, such as tumor spheroids, is burdensome since it often entails the collection of a stack of multiple optical sections along the z-dimension, which is time-consuming and has high risk of causing phototoxicity. To alleviate this problem, we propose a workflow combining axial z-sweep acquisition and deep learning-based image enhancement. This paper is novel and the contributions are good for a journal article. The revised version of the paper may be considered for publication in this journal.

1. The literature of the paper is poor, the authors may be consider the most recent articles for literature.
Author response: We have added the information in **bold** in the Introduction:
"Whereas traditional PSF-based image restoration is incredibly challenging for complex 3D samples, convolutional neural networks (CNNs) are now routinely used for such difficult computer vision problems. Across various 3D fluorescence imaging studies, CNNs are used to segment nuclei [11, 12] and cell bodies [13, 14], image restoration [15, 16], image super-resolution [17, 18], as well as blind deconvolution in 2D widefield fluorescence imaging [19, 20]. **In the 3D fluorescence imaging space, recent studies have outlined methods to use CNNs for virtual refocusing to predict a user-defined 3D surface from a single plane 2D fluorescent image [21, 22], using recurrent neural networks (RNNs) to reconstruct a complete 3D volume with far fewer optical sections than typically needed [23], and optimization of phase mask filters for PSF-engineering to capture 2D extended depth-of-field images or 3D volumetric images [24, 25]. While these notable advancements demonstrate the potential of CNNs to increase throughput for 3D fluorescent imaging, they have primarily been limited to high-resolution, subcellular imaging. These studies do not address the need for improving throughput capabilities for imaging large 3D models, such as tumor spheroids and organoids, which can be larger than a millimeter in diameter. Therefore, there exists a gap in the field whereby high-throughput fluorescent imaging of large, complex 3D samples can be accomplished without complicated hardware or acquiring multiple z-slices of samples.**"

2. The reasons to achieve the superior performance of the article may be included in the revised version of the article.
Author response: We have clarified the advantage of our approach in the first paragraph of the discussion:
"**...Notably, for our embedded tumor multi-spheroid samples, which required acquisition of data across a 1.5mm volume, the conventional z-stack approach took 101 seconds for a single sample whereas the z-sweep only took 1.2 seconds, nearly achieving a 100-fold speedup...**"
We have also expanded on the reasons why neural networks are suitable for this task in the third paragraph of the discussion as:
"**Regardless of the neural network architecture used, images that result from a z-sweep acquisition are well-suited for a deep learning based restoration workflow. While indeed appearing low in quality, z-sweep images effectively represent the fluorescent intensity of the sample integrated over the z-axis. Therefore, we speculate that the**

z-sweep sufficiently provides a neural network with enough real information about the sample to accurately predict the restored unintegrated fluorescent signal."

3. List the limitations of the proposed work.
Author response: The limitations of the proposed work is expanded on in comment 2 above where the acquisition speed is 1.2 seconds per sample. The almost 100-fold speedup compared to the z-stack approach is a big step in the right direction but still requires 1.6 minutes per 96 well plate. This is enough for a significantly higher throughput but one can always aim for even faster acquisition in the future. Additionally, in comment 4 below we expand on the post processing of the images with the proposed work. Furthermore, we have trained the model on one of the three common types of experimental preparations for culturing spheroids we consider and have noted a transferability across these three different cell cultures. This is surely promising for further exploration of the z-sweep approach on other types of cell cultures, FPs, organoids etc. but not a guarantee. This is also discussed in the Discussion.

4. Discuss the complexity of the proposed model and how efficient it is over the existing ones.
Author response: A z-sweep is acquired in 1.2 seconds compared to the 101 seconds a z-stack takes to acquire resulting in an almost 100-fold speedup. Therefore, the big advantage in our approach is the acquisition of the images. However, the trained DL-model does not cause a bottleneck either. An OSA-CGAN model evaluates a z-sweep image in 0.33s on a laptop equipped with an NVIDIA Quadro RTX 3000 and requires 4015MiB VRAM. Running the same model on an Intel Core i7-CPU @ 2.30GHz requires 10s per image.

5. Provide the dataset details or citations.
Author response: Thank you for acknowledging this. We have added a sentence in the Experiment section where we point the reader figshare `https://figshare.com/projects/Dataset_of_fluorecent_3D-samples_projected_to_2D/126629` where the data is available.

6. There are several performance metrics in the literature, whereas the authors consider only few in the paper.
Author response: We have considered two additional metrics from the literature which we consider relevant. MS-SSIM to extend the analysis on multiple scale and capture information about details from multiple resolutions and MSE since it's one of the most common metrics to asses the difference of individual pixels. These are added to Table 1 in the results as well as discussed in the Discussion.

References

[1] Magidson V, Khodjakov A. Circumventing photodamage in live-cell microscopy. Methods in Cell Biology. 2013;114:545–560.

[2] Elliott AD. Confocal microscopy: principles and modern practices. Current protocols in cytometry. 2020;92(1):e68.

[3] Bayguinov PO, Oakley DM, Shih CC, Geanon DJ, Joens MS, Fitzpatrick JA. Modern laser scanning confocal microscopy. Current protocols in cytometry. 2018;85(1):e39.

[4] Chang BJ, Manton JD, Sapoznik E, Pohlkamp T, Terrones TS, Welf ES, et al. Real-time multi-angle projection imaging of biological dynamics. Nature Methods. 2021;18(7):829–834. doi:10.1038/s41592-021-01175-7.

[5] Carpenter AE, Jones TR, Lamprecht MR, Clarke C, Kang IH, Friman O, et al. CellProfiler: image analysis software for identifying and quantifying cell phenotypes. Genome biology. 2006;7(10):1–11.

[6] Schindelin J, Arganda-Carreras I, Frise E, Kaynig V, Longair M, Pietzsch T, et al. Fiji: an open-source platform for biological-image analysis. Nature methods. 2012;9(7):676–682.

[7] Duval K, Grover H, Han LH, Mou Y, Pegoraro AF, Fredberg J, et al. Modeling Physiological Events in 2D vs. 3D Cell Culture. Physiology. 2017;32(4):266–277. doi:10.1152/physiol.00036.2016.

[8] Kapałczyńska M, Kolenda T, Przybyła W, Zajączkowska M, Teresiak A, Filas V, et al. 2D and 3D cell cultures – a comparison of different types of cancer cell cultures. Archives of Medical Science. 2018;14(4):910–919. doi:10.5114/aoms.2016.63743.

[9] Chen BC, Legant WR, Wang K, Shao L, Milkie DE, Davidson MW, et al. Lattice light-sheet microscopy: Imaging molecules to embryos at high spatiotemporal resolution. Science. 2014;346(6208). doi:10.1126/science.1257998.

[10] Woringer M, Darzacq X, Zimmer C, Mir M. Faster and less phototoxic 3D fluorescence microscopy using a versatile compressed sensing scheme. Optics express. 2017;25(12):13668–13683.

[11] Joon Ho D, Fu C, Salama P, Dunn KW, Delp EJ. Nuclei segmentation of fluorescence microscopy images using three dimensional convolutional neural networks. In: Proceedings of the IEEE Conference on Computer Vision and Pattern Recognition Workshops; 2017. p. 82–90.

[12] Ho DJ, Fu C, Salama P, Dunn KW, Delp EJ. Nuclei detection and segmentation of fluorescence microscopy images using three dimensional convolutional neural networks. In: 2018 IEEE 15th International Symposium on Biomedical Imaging (ISBI 2018). IEEE; 2018. p. 418–422.

[13] Stegmaier J, Spina TV, Falcao AX, Bartschat A, Mikut R, Meyerowitz E, et al. Cell segmentation in 3D confocal images using supervoxel merge-forests with CNN-based hypothesis selection. In: 2018 IEEE 15th International Symposium on Biomedical Imaging (ISBI 2018). IEEE; 2018. p. 382–386.

[14] Castelli FM, Roffilli M, Mazzamuto G, Costantini I, Silvestri L, Pavone FS. Semantic Segmentation of Neuronal Bodies in Fluorescence Microscopy Using a 2D+ 3D CNN Training Strategy with Sparsely Annotated Data. In: International Conference on Machine Learning, Optimization, and Data Science. Springer; 2020. p. 95–99.

[15] Weigert M, Royer L, Jug F, Myers G. Isotropic reconstruction of 3D fluorescence microscopy images using convolutional neural networks. In: International Conference on Medical Image Computing and Computer-Assisted Intervention. Springer; 2017. p. 126–134.

[16] Weigert M, Schmidt U, Boothe T, Müller A, Dibrov A, Jain A, et al. Content-aware image restoration: pushing the limits of fluorescence microscopy. Nature Methods. 2018;15(12):1090–1097.

[17] Zhang H, Fang C, Xie X, Yang Y, Mei W, Jin D, et al. High-throughput, high-resolution deep learning microscopy based on registration-free generative adversarial network. Biomed Opt Express. 2019;10(3):1044–1063. doi:10.1364/BOE.10.001044.

[18] Wang H, Rivenson Y, Jin Y, Wei Z, Gao R, Günaydın H, et al. Deep learning enables cross-modality super-resolution in fluorescence microscopy. Nature methods. 2019;16(1):103–110.

[19] Lim S, Ye JC. Blind deconvolution microscopy using cycle consistent CNN with explicit PSF layer. In: International Workshop on Machine Learning for Medical Image Reconstruction. Springer; 2019. p. 173–180.

[20] Lee S, Han S, Salama P, Dunn KW, Delp EJ. Three dimensional blind image deconvolution for fluorescence microscopy using generative adversarial networks. In: 2019 IEEE 16th International Symposium on Biomedical Imaging (ISBI 2019). IEEE; 2019. p. 538–542.

[21] Wu Y, Rivenson Y, Wang H, Luo Y, Ben-David E, Bentolila LA, et al. Three-dimensional virtual refocusing of fluorescence microscopy images using deep learning. Nature methods. 2019;16(12):1323–1331.

[22] Wu Y, Rivenson Y, Wang H, Luo Y, Ben-David E, Bentolila LA, et al. Deep-Z: 3D Virtual Refocusing of Fluorescence Images Using Deep Learning. In: CLEO: Applications and Technology. Optical Society of America; 2020. p. AW3T–6.

[23] Huang L, Chen H, Luo Y, Rivenson Y, Ozcan A. Recurrent neural network-based volumetric fluorescence microscopy. Light: Science & Applications. 2021;10(1):1–16.

[24] Nehme E, Freedman D, Gordon R, Ferdman B, Weiss LE, Alalouf O, et al. DeepSTORM3D: dense 3D localization microscopy and PSF design by deep learning. Nature Methods. 2020;17(7):734–740.

[25] Page J, Favaros P. Learning to Model and Calibrate Optics Via a Differentiable Wave Optics Simulator. In: 2020 IEEE International Conference on Image Processing (ICIP). IEEE; 2020. p. 2995–2999.

[26] Ronneberger O, Fischer P, Brox T. U-net: Convolutional networks for biomedical image segmentation. In: International Conference on Medical image computing and computer-assisted intervention. Springer; 2015. p. 234–241.

[27] Lee Y, Park J. CenterMask: Real-Time Anchor-Free Instance Segmentation. In: 2020 IEEE/CVF Conference on Computer Vision and Pattern Recognition (CVPR); 2020. p. 13903–13912.

[28] Hu J, Shen L, Sun G. Squeeze-and-excitation networks. In: Proceedings of the IEEE conference on computer vision and pattern recognition; 2018. p. 7132–7141.

[29] Isola P, Zhu JY, Zhou T, Efros AA. Image-to-Image Translation with Conditional Adversarial Networks. In: 2017 IEEE Conference on Computer Vision and Pattern Recognition (CVPR); 2017. p. 5967–5976.

[30] Salimans T, Goodfellow I, Zaremba W, Cheung V, Radford A, Chen X, et al. Improved Techniques for Training GANs. In: Lee D, Sugiyama M, Luxburg U, Guyon I, Garnett R, editors. Advances in Neural Information Processing Systems. vol. 29. Curran Associates, Inc.; 2016.

[31] Goodfellow IJ, Pouget-Abadie J, Mirza M, Xu B, Warde-Farley D, Ozair S, et al. Generative Adversarial Networks. arXiv:14062661 [cs, stat]. 2014;.

[32] Costa EC, Moreira AF, de Melo-Diogo D, Gaspar VM, Carvalho MP, Correia IJ. 3D tumor spheroids: an overview on the tools and techniques used for their analysis. Biotechnology advances. 2016;34(8):1427–1441.

[33] ; Image Processing and Segmentation of Sets of Z-Stacked Images of Three-Dimensional Biological Samples. US20210327080A1; April 21, 2020.

[34] Forster B, Van De Ville D, Berent J, Sage D, Unser M. Extended depth-of-focus for multi-channel microscopy images: a complex wavelet approach. In: 2004 2nd IEEE International Symposium on Biomedical Imaging: Nano to Macro (IEEE Cat No. 04EX821). IEEE; 2004. p. 660–663.

[35] Kingma DP, Ba JL. Adam: A method for stochastic gradient descent. In: ICLR: International Conference on Learning Representations; 2015. p. 1–15.

[36] Forsgren E. ProjSweep; 2021. `https://github.com/edvinforsgren/ProjSweep`.

[37] Heusel M, Ramsauer H, Unterthiner T, Nessler B, Hochreiter S. GANs Trained by a Two Time-Scale Update Rule Converge to a Local Nash Equilibrium. In: Guyon I, Luxburg UV, Bengio S, Wallach H, Fergus R, Vishwanathan S, et al., editors. Advances in Neural Information Processing Systems. vol. 30. Curran Associates, Inc.; 2017.

[38] Davoudi N, Deán-Ben XL, Razansky D. Deep learning optoacoustic tomography with sparse data. Nature Machine Intelligence. 2019;1(10):453–460.

[39] Zhang H, Wu C, Zhang Z, Zhu Y, Lin H, Zhang Z, et al. Resnest: Split-attention networks. arXiv preprint arXiv:200408955. 2020;.

[40] Huang G, Liu Z, Van Der Maaten L, Weinberger KQ. Densely connected convolutional networks. In: Proceedings of the IEEE conference on computer vision and pattern recognition; 2017. p. 4700–4708.

[41] Wang X, Yan H, Huo C, Yu J, Pant C. Enhancing Pix2Pix for remote sensing image classification. In: 2018 24th International Conference on Pattern Recognition (ICPR). IEEE; 2018. p. 2332–2336.

[42] Salehi P, Chalechale A. Pix2pix-based stain-to-stain translation: A solution for robust stain normalization in histopathology images analysis. In: 2020 International Conference on Machine Vision and Image Processing (MVIP). IEEE; 2020. p. 1–7.

[43] Mori M, Fujioka T, Katsuta L, Kikuchi Y, Oda G, Nakagawa T, et al. Feasibility of new fat suppression for breast MRI using pix2pix. Japanese Journal of Radiology. 2020;38(11):1075–1081.
